# Preoperative prediction of glypican-3 positive expression in solitary hepatocellular carcinoma on gadoxetate-disodium enhanced magnetic resonance imaging

**DOI:** 10.3389/fimmu.2022.973153

**Published:** 2022-08-25

**Authors:** Yidi Chen, Yun Qin, Yuanan Wu, Hong Wei, Yi Wei, Zhen Zhang, Ting Duan, Hanyu Jiang, Bin Song

**Affiliations:** ^1^ Department of Radiology, West China Hospital, Sichuan University, Chengdu, China; ^2^ Big Data Research Center, University of Electronic Science and Technology of China, Chengdu, China; ^3^ Department of Radiology, Sanya People’s Hospital, Sanya, China

**Keywords:** magnetic resonance imaging, glypican-3, hepatocellular carcinoma, immunotherapy, diagnosis

## Abstract

**Purpose:**

As a coreceptor in Wnt and HGF signaling, glypican-3 (GPC-3) promotes the progression of tumor and is associated with a poor prognosis in hepatocellular carcinoma (HCC). GPC-3 has evolved as a target molecule in various immunotherapies, including chimeric antigen receptor T cell. However, its evaluation still relies on invasive histopathologic examination. Therefore, we aimed to develop an easy-to-use and noninvasive risk score integrating preoperative gadoxetic acid–enhanced magnetic resonance imaging (EOB-MRI) and clinical indicators to predict positive GPC-3 expression in HCC.

**Methods and materials:**

Consecutive patients with surgically-confirmed solitary HCC who underwent preoperative EOB-MRI between January 2016 and November 2021 were retrospectively included. EOB-MRI features were independently evaluated by two masked abdominal radiologists and the expression of GPC-3 was determined by two liver pathologists. On the training dataset, a predictive scoring system for GPC-3 was developed against pathology *via* logistical regression analysis. Model performances were characterized by computing areas under the receiver operating characteristic curve (AUCs).

**Results:**

A total of 278 patients (training set, n=156; internal validation set, n=39; external validation set, n=83) with solitary HCC (208 [75%] with positive GPC-3 expression) were included. Serum alpha-fetoprotein >10 ng/ml (AFP, odds ratio [OR]=2.3, four points) and five EOB-MR imaging features, including tumor size >3.0cm (OR=0.5, -3 points), nonperipheral “washout” (OR=3.0, five points), infiltrative appearance (OR=9.3, 10 points), marked diffusion restriction (OR=3.3, five points), and iron sparing in solid mass (OR=0.2, -7 points) were significantly associated with positive GPC-3 expression. The optimal threshold of scoring system for predicting GPC-3 positive expression was 5.5 points, with AUC 0.726 and 0.681 on the internal and external validation sets, respectively.

**Conclusion:**

Based on serum AFP and five EOB-MRI features, we developed an easy-to-use and noninvasive risk score which could accurately predict positive GPC-3 HCC, which may help identify potential responders for GPC-3-targeted immunotherapy.

## Introduction

Primary liver cancer is the sixth most commonly diagnosed cancer and the third leading cause of cancer-related death worldwide, and hepatocellular carcinoma (HCC) comprises 75% to 85% of cases (1). Immunotherapies play an increasingly central role in the management of HCC, among which chimeric antigen receptor (CAR) T cell therapy is regarded as a promising next-generation immunotherapy regimen with remarkable safety and efficacy profiles (2). However, CAR T cell therapy was shown to provide clinical benefit in limited HCC patients, possibly due to a lack of tumor specific antigens and an immunosuppressive tumor microenvironment (3). Therefore, there remains an unmet need for biomarker discovery which could help identify potential responders and direct individualized treatment decision-making.

In HCC, Wnt signaling has been reported to promote hepatocarcinogenesis, tumor growth and dissemination, while the hepatocyte growth factor (HGF) is also associated with increased hepatocarcinogenesis and metastasis. Glypican-3 (GPC-3) is a heparan sulfate glycoprotein which serves as a coreceptor in Wnt and HGF signaling. In specific, GPC-3 binds to the cell membrane and is involved in organ morphogenesis by regulating cell proliferation through modulation of Wnt signaling. Moreover, it is also involved in HCC cell migration and motility through heparan sulfate chain-mediated cooperation with the HGF/Met pathway (4, 5). Therefore, it has also been reported to promote tumor progression and associated with a poor prognosis in HCC (6–8). Currently, GPC-3 is widely used for diagnostic purposes because of it is specifically expressed in around 70–80% of HCCs (9). More recently, GPC-3 has gained much attention as a novel target molecule in immunotherapies. In specific, Shi D et al. (10) firstly published a phase I trial of CAR-GPC-3 T-cell therapy in patients with advanced HCC, and their results demonstrated the initial safety profile and early signs of antitumor activity of CAR-GPC-3 T cells. In addition, Liu et al. (5) reported that a novel human monoclonal antibody (32A9), as a GPC-3-specifc antibody which efficiently eliminated GPC3-positive HCC cells *in vitro* and induced HCC xenograft tumor regressions *in vivo*. Comparison with traditional therapies, tumor antigen-targeting antibody- and immune modulating antibody-based immunotherapies represent emerging approaches that may improve HCC treatment outcomes (11). Therefore, GPC-3 holds potential to serve as an effective biomarker for patient selection in HCC immunotherapy. However, the assessment of GPC-3 expression still mandates invasive histopathologic examination, which is sensitive to sampling errors and not routinely performed in the clinical practice.

Magnetic resonance imaging (MRI) could be used for visualization of specific target or drug delivery carrier accumulation in tumors (12, 13). Similarly, GPC-3 expression can be evaluated *via* noninvasive imaging techniques. For example, MRI specific superparamagnetic iron oxide (SPIO) anti-GPC-3 molecular probe has demonstrated the effectiveness for assessing GPC-3 expression in HCC tissues (14–16), and a few studies revealed the associations between GPC-3 expression with various MRI morphological features and quantitative parameters (e.g., MRI-based radiomics and iterative decomposition of water and fat with echo asymmetry and least squares estimation [IDEAL-IQ] parameters) (17–19). Despite promising results so far, existing evidence were not yet ready to be transformed into routine practice due to the limited application of SPIO and poor interpretability of quantitative techniques.

Therefore, in patients who underwent curative-intent surgery for solitary HCC, this study aimed to develop and validate a simple, noninvasive, and interpretable model to predict GPC-3 expression based on gadoxetic acid (EOB)-enhanced MRI and clinical features and to explore the model’s efficacy in stratifying postoperative survival.

## Materials and methods

This retrospective study was approved by the Ethics Committee of West China Hospital, Sichuan University (Chengdu, China), and the requirements to obtain written informed consent were waived (Approved No. 2022-651).

### Patients

**Figure 1:**
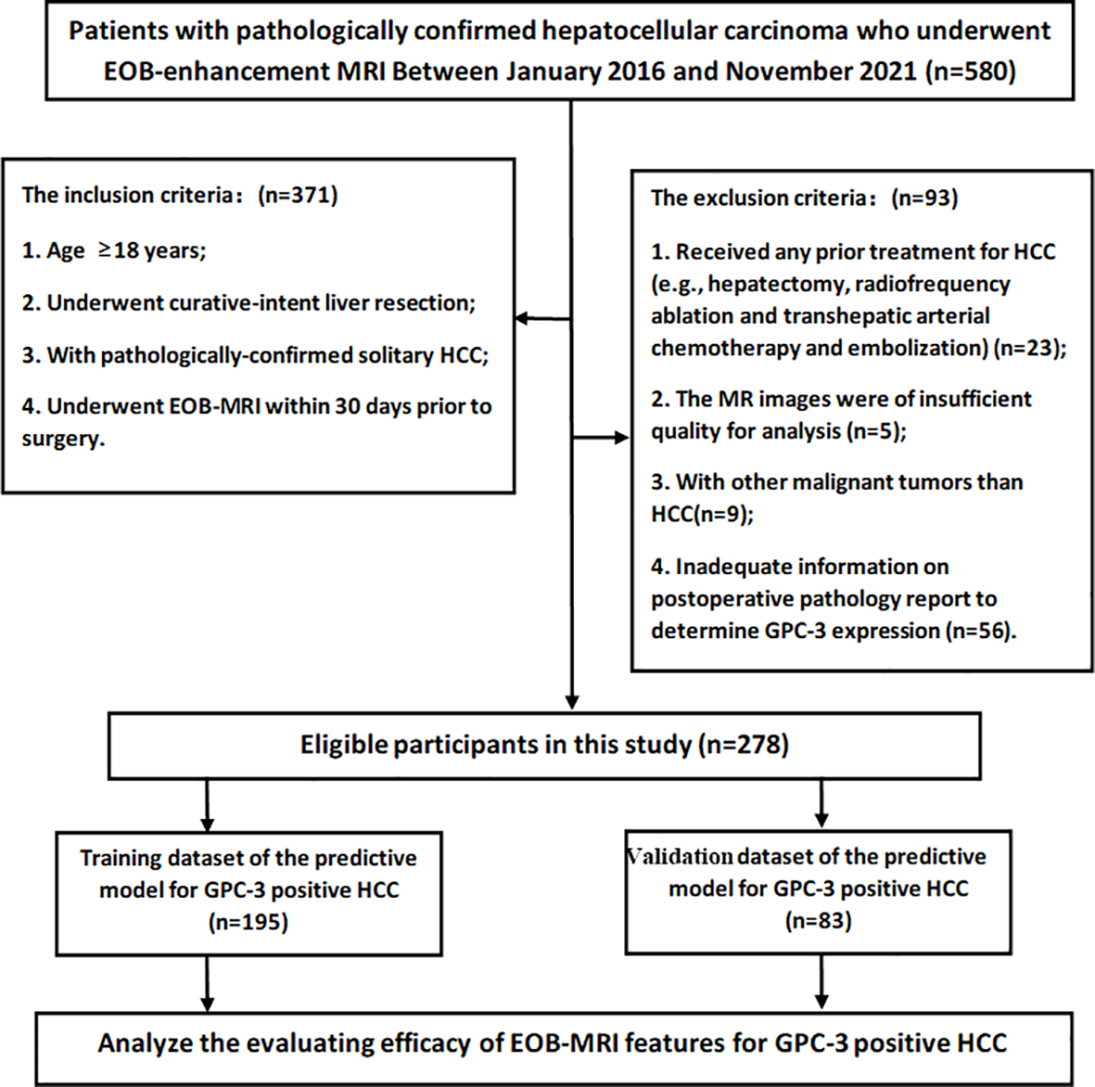
The flowchart of the retrospective study cohort. A total of 278 participants with solitary hepatocellular carcinoma were included in this study.

Between January 2016 and November 2021, consecutive patients who fulfilled the following inclusion criteria were enrolled: (a) age ≥18 years;(b) underwent curative-intent liver resection; (c) with pathologically-confirmed solitary HCC; and (d) underwent EOB-MRI within 30 days prior to surgery. The exclusion criteria were as follows: (a) received any prior treatment for HCC (e.g., hepatectomy, radiofrequency ablation and transhepatic arterial chemotherapy and embolization); (b) the MR images were of insufficient quality for analysis (e.g., severe artifact); (c) with other malignant tumors than HCC (n=9); and (d) inadequate information on postoperative pathology report to determine GPC-3 expression. Detailed patient inclusion and exclusion are illustrated in **Figure 1**.

Baseline clinical information, including patient demographics, causes of liver diseases, and key laboratory test results (alpha-fetoprotein [AFP], aspartate aminotransferase [AST], alanine aminotransferase [ALT] and serum total bilirubin [TBIL]) were collected from electronic medical records.

### MRI acquisition and analysis

Four 3.0 T MR scanners (Discovery 750, SIGNA™ Architect, and SIGNA™ Premier, GE Healthcare; and MAGNETOM Skyra, Siemens Healthineers) and one 1.5 T MRI scanner (uMR588, United Imaging Healthcare) were used to acquire MR images. The sequences included: T2-weighted imaging, diffusion-weighted imaging with apparent diffusion coefficient maps, T1-weighted in-phase and opposed-phase imaging, and T1-weighted dynamic imaging with gadoxetic acid disodium (Primovist^®^, Bayer Pharma AG). Details of the MRI technique are provided in Supplementary A1 and **Table S1**.

Two fellowship-trained abdominal radiologists (with 8 and 6 years of experiences in liver MR imaging, respectively) who were blinded of the clinical, laboratory, histopathologic and follow-up information independently reviewed all MR images. Any discrepancy in imaging interpretation was resolved by a third radiologist who had over 20 years of experience in liver MR imaging.

The reviewers evaluated the presence/absence of a total of 37 imaging features which were reported to associate with the underlying liver disease (e.g., radiologically-evident cirrhosis), tumor burden (e.g., bilobar involvement), tumor extent (e.g., lymph node metastasis, macrovascular invasion), tumor aggressiveness (e.g., intratumoral artery, non-smooth tumor margin, peritumoral hepatobiliary phase hypointensity) and the Liver Imaging Reporting and Data System (LI-RADS) version 2018 features and categories. Definitions of the evaluated imaging features are summarized in **Table S2**.

### Reference standard

Information on GPC-3 expression, as documented by another radiologist without knowing the patient’s imaging and clinical data, was retrieved from routine pathological reports as the reference standard in the current study.

In specific, complete HCC specimens were obtained after hepatectomy, and a standard seven-point sampling method (20) was used to assess tumor and peritumoral pathologic features. To accurately evaluate the expression of GPC-3, we adopted the scoring scale proposed by Takai et al. (21) which took into account positive cell rate, staining intensity, and staining pattern. Based on this scoring scale, the positive cell rate was graded from 0 to 3+ as 0 (<5% tumor cells positive), 1+ (5–10% tumor cells positive), 2+ (10–50% tumor cells positive), and 3+ (>50% tumor cells positive). The staining intensity was classified as weak, moderate, and strong staining. The staining pattern was graded on a scale of I–III based on whether the cell membrane manifested as incomplete (I: globally incomplete; II: generally incomplete with some complete staining) or complete circumferential staining (III: generally complete with some incomplete staining). In the current study, grade 0 positive cell rate with any staining intensity or grade 1+ positive cell rate with weak staining were regarded as GPC-3 negative.

### Patient follow-up

Patients underwent routine postoperative follow-up at 1 month, every 3 months for the first 2 years and every 6 months thereafter with serum AFP and contrast-enhanced imaging modality (ultrasound, CT, or MRI). Tumor recurrence was confirmed by imaging or pathologic examinations during follow-up. Recurrence-free survival (RFS) was defined as the time from the date of surgery to that of tumor recurrence or the last follow-up date (May 1, 2022), whichever occurred first. Overall survival (OS) was defined as the time from the date of surgery to that of death by any cause, or the last follow-up date, whichever occurred first.

### Statistical analysis

Differences in continuous variables were compared using either the Student’s t test or the Mann-Whitney U test, while those in categorical variables were investigated by either the chi-square test or the Fisher’s exact test, as appropriate.

#### Assessment for inter-rater agreement

Inter-rater agreement between the two reviewers were assessed by calculating the intraclass correlation coefficient for continuous variables, Cohen’s κ values for binary variables and weighted κ values for ordinal/categorical variables, respectively.

#### Development and validation of the predictive model for GPC-3

On a per-patient basis, 70% of randomly selected patients (n=195) were assigned into the training dataset, while the remaining 30% (n=83) into an independent external validation dataset.

On the training dataset, clinical and imaging predictors for GPC-3 were selected *via* univariable logistic regression analysis. To improve clinical utility and model simplicity, continuous variables were converted to categorical or dichotomized variables according to ranges of normality or clinical relevance (showed in **Table 2**). Thereafter, all predictors with *P* values < 0.1 were fit into a multivariable logistic regression model with backward stepwise method and 5-fold cross validation (creating the “internal validation” dataset) while controlled for patient age, gender, and HBV infection status (infected vs. non-infected). The Akaike Information Criterion was used to obtain the most parsimonious feature combination. A scoring system and nomogram was constructed based on the significant predictors at multivariable regression analysis to estimate the probability of GPC-3 expression, and the optimal threshold of the nomogram was determined by receiver operating characteristic analysis with the Youden’s index.

On the external validation dataset, the diagnostic performances of the predictive model for assessing GPC-3 expression were evaluated by computing area under the receiver operating characteristic curve (AUC), sensitivity, specificity, positive predictive value (PPV), negative predictive value (NPV), and accuracy. Calibration curves were plotted to investigate the model calibration by the Hosmer-Lemeshow test, and decision curve analysis was conducted to estimate the model’s clinical usefulness by quantifying the net benefits at different threshold probabilities. Survival outcomes were evaluated using the Kaplan-Meier method and compared with the log-rank test.

All statistical analyses were conducted with R software (version 3.5.1; http://www.Rproject.org) and SPSS (version 22.0). A two-sided p<0.05 was used to indicate statistically significant difference.

## Results

### Patients

A total of 278 eligible patients (222 male; 53.6 ± 11.6 years) were enrolled during the study period (**Figure 1**), 208 (74.8%) with GPC-3 positive HCC and 70 (25.2%) with GPC-3 negative HCC. In the GPC-3 positive and negative groups, the mean patient age was 52.9 ± 11.7 and 55.8 ± 11.3 years, respectively (*P*=0.066); male and female patients were 165 (79.3%) and 43 (20.7%) in positive group, 57 (81.4%) and 13 (18.6%) in negative group (*P*=0.735); the number of patients with hepatitis B was 193 (92.8%) and 60 (85.7%), respectively, the cause of liver disease was no significant difference (*P*=0.304); the median of serum AFP was 40.6 (range 0.98–25451) ng/mL and 6.73 (range 1.0~2484.0) ng/mL, respectively *(P*=0.009); serum AST was 29.0 (13.0~723.0) U/L and 35.0 (13.0~243.0) U/L, respectively (*P*=0.043); serum ALB was 43.1 (30.6~66.5) g/L and 41.9 (27.2~51.0) g/L, respectively (*P*=0.043); no significant difference in other clinical characteristics was detected between the GPC-3 positive and negative groups. (**Table 1**)

**Table 1:** The clinical characteristics of patients with HCC.

Variables	All patients	GPC-3 positive (n = 208)	GPC-3 negative (n = 70)	*P* value
Age (year)	53.9 ± 11.7	52.9 ± 11.7	55.8 ± 11.3	0.066
Sex (n, %)				0.735
Male	222 (79.9%)	165 (79.3%)	57 (81.4%)	
Female	56 (20.1%)	43 (20.7%)	13 (18.6%)	
Cause of liver disease				0.304
HBV	253 (91.0%)	193 (92.8%)	60 (85.7%)	
HCV	3 (1.1%)	2 (1.0%)	1 (1.4%)	
HBV+HCV	4 (1.4%)	3 (1.4%)	1 (1.4%)	
Alcohol	2 (0.7%)	1 (0.5%)	1 (1.4%)	
NAFLD	5 (1.8%)	3 (1.4%)	2 (2.9%)	
Autoimmune disease	11 (4.0%)	6 (2.9%)	5 (7.1%)	
Cirrhosis				0.263
Presence	161 (57.9%)	116 (55.8%)	45 (64.3%)	
Absence	117 (42.1%)	92 (44.2%)	25 (35.7%)	
BCLC stage				0.074
0	79 (28.4%)	66 (31.7%)	13 (18.6%)	
A	184 (66.2%)	130 (62.5%)	54 (77.1%)	
C	15 (5.4%)	12 (5.8%)	3 (4.3%)	
AFP (ng/mL)	21.2 (1.0~50143.0)	40.6 (1.0~50143.0)	6.73 (1.0~2484.0)	0.009
PIVKA.II (AU/ml)	75.5 (0~75000)	77.0 (0~75000.0)	65.0 (11.0~75000.0)	0.428
CEA (ng/ml)	2.14 (1.0~302)	2.07 (1.0~302.0)	2.48 (1.0~10.0)	0.126
CA199 (U/ml)	16.3 (1~596.0)	15.9 (1.0~596.0)	19.5 (1.0~253.0)	0.080
TBIL (μmol/L)	13.9 (5.6~56.5)	14.2 (5.9~56.5)	13.1 (5.6~35.5)	0.520
ALT (U/L)	30.0 (6.0~1010.0)	30.0 (8.0~1010.0)	38.0 (6.0~215.0)	0.066
AST (U/L)	29.0 (13.0~723.0)	29.0 (13.0~723.0)	35.0 (13.0~243.0)	0.043
ALB (U/L)	42.7 (27.2~66.5)	43.1 (30.6~66.5)	41.9 (27.2~51.0)	0.043
PLT (10^9^/L)	126.0 (5.0~470.0)	129.0 (5.0~410.0)	116.0 (25.0~470.0)	0.503

HBV, hepatitis B virus; HCV, hepatitis C virus; NAFLD, non-alcoholic fatty liver disease; BCLC stage, Barcelona clinic liver cancer stage; AFP, alpha-fetoprotein; PIVKA.II, protein induced by vitamin-K absence or antagonist II; CEA, carcinoma embryonic antigen; TBIL, total bilirubin; ALT, alanine transaminase; AST, aspartate aminotransferase; ALB, serum albumin; PLT, platelet count.

No difference in baseline clinical features was detected between the training and validation datasets (*P* > 0.05 for all; **Supplementary Table S3**).

### Correlations between EOB-MRI features and GPC-3 expression

In the groups of GPC-3 positive and negative HCC, the tumor size were 3.73 ± 2.7 cm and 4.4 ± 2.8 cm, respectively (*P*=0.091); the number of patients presenting nonperipheral “washout” were 182 (87.5%) and 65 (92.9%), respectively (*P*=0.095); the number of patients presenting “infiltrative appearance” were 28 (13.5%) and three (4.3%), respectively (*P*=0.046); the number of patients presenting “marked diffusion restriction” were 87 (41.8%) and 17 (24.3%), respectively (*P*=0.01); the number of patients presenting “iron sparing in solid mass” were 34 (16.3%) and 18 (25.7%), respectively (*P*=0.11). The number of patients with LI-RADS categories of 4, 5 and M were 15 (7.2%), 175 (84.1%),18 (8.7%) for patients with positive GPC-3 expressions, and three (4.3%), 64 (91.4%), three (4.3%) for patients with negative GPC-3 expressions, respectively (*P*=0.399); no significant difference in other EOB-MRI features was detected between the GPC-3 positive and negative groups. (**Table 2**)

**Table 2:** The MRI features and consistency analysis between two viewers.

Variables	GPC-3 positive (n = 208)	GPC-3 negative (n = 70)	P^*^ value	Kappa value	*P^#^* value
LI_RADS			0.399	0.498	<0.001
4	15 (7.2%)	3 (4.3%)			
5	175 (84.1%)	64 (91.4%)			
M	18 (8.7%)	3 (4.3%)			
Size (cm)	3.73 ± 2.7	4.4 ± 2.8	0.091	–	–
APHE			0.488	0.458	<0.001
Presence	201 (96.6%)	70 (100%)			
Absence	7 (3.4%)	0 (0.0%)			
Internal artery			0.26	0.518	<0.001
Presence	47 (22.6%)	21 (30.0%)			
Absence	161 (77.4%)	49 (70.0%)			
Corona enhancement			0.453	0.346	<0.001
Presence	64 (30.8%)	18 (25.7%)			
Absence	144 (69.2%)	52 (74.3%)			
Nonperipheral “washout”			0.095	0.400	<0.001
Presence	182 (87.5%)	65 (92.9%)			
Absence	26 (12.5%)	5 (7.1%)			
Complete capsule			1.00	0.409	<0.001
Presence	95 (45.7%)	32 (45.7%)			
Absence	113 (54.3%)	38 (54.3%)			
Blood products in mass			1.00	0.649	<0.001
Presence	58 (27.9%)	19 (27.1%)			
Absence	150 (72.1%)	51 (72.9%)			
Nodule in nodule			0.36	0.200	0.001
Presence	56 (26.9%)	23 (32.9%)			
Absence	152 (73.1%)	47 (67.1%)			
Mosaic architecture			0.363	0.261	<0.001
Presence	58 (27.9%)	24 (34.3%)			
Absence	150 (72.1%)	46 (65.7%)			
Infiltrative appearance			0.046	0.430	<0.001
Presence	28 (13.5%)	3 (4.3%)			
Absence	180 (86.5%)	67 (95.7%)			
Necrosis or severe ischemia			0.260	0.547	<0.001
Presence	47 (22.6%)	21 (30.0%)			
Absence	161 (77.4%)	49 (70.0%)			
Tumor margin			0.273	0.416	<0.001
Smooth	97 (46.6%)	38 (54.3%)			
Non-smooth	111 (53.4%)	32 (45.7%)			
Marked diffusion restriction			0.01	0.387	<0.001
Presence	87 (41.8%)	17 (24.3%)			
Absence	121 (58.2%)	53 (75.7%)			
Marked T2 hyperintense			1.00	0.436	<0.001
Presence	4 (1.9%)	1 (1.4%)			
Absence	204 (98.1%)	69 (98.6%)			
Fat in mass more than liver			0.037	0.268	<0.001
Presence	102 (49.0%)	24 (34.3%)			
Absence	106 (51.0%)	46 (65.7%)			
Fat sparing in solid mass			0.402	0.438	<0.001
Presence	16 (7.7%)	4 (5.7%)			
Absence	192 (92.3%)	56 (94.3%)			
Iron in mass more than liver			0.373	0.378	<0.001
Presence	4 (1.9%)	3 (4.3%)			
Absence	204 (98.1%)	67 (95.7%)			
Iron sparing in solid mass			0.110	0.432	<0.001
Presence	34 (16.3%)	18 (25.7%)			
Absence	174 (83.7%)	52 (74.3%)			
HBP hypointense			0.007	0.471	<0.001
Presence	204 (98.1%)	63 (90.0%)			
Absence	4 (1.9%)	7 (10.0%)			
HBP Peritumoral hypointense			1.00	0.507	<0.001
Presence	67 (32.2%)	23 (32.9%)			
Absence	141 (67.8%)	47 (67.1%)			
Targetoid TP or HBP appearance			1.00	0.122	0.010
Presence	2 (1.0%)	0 (0.0%)			
Absence	206 (99.0%)	70 (100%)			

APHE, arterial phase hyperenhancement; TP, Transitional phase; HBP, hepatobiliary phase.

P* values correspond to the MRI features, and P^#^ values correspond to the Kappa values.

### Development of the GPC-3 prediction model

**Figure 2:**
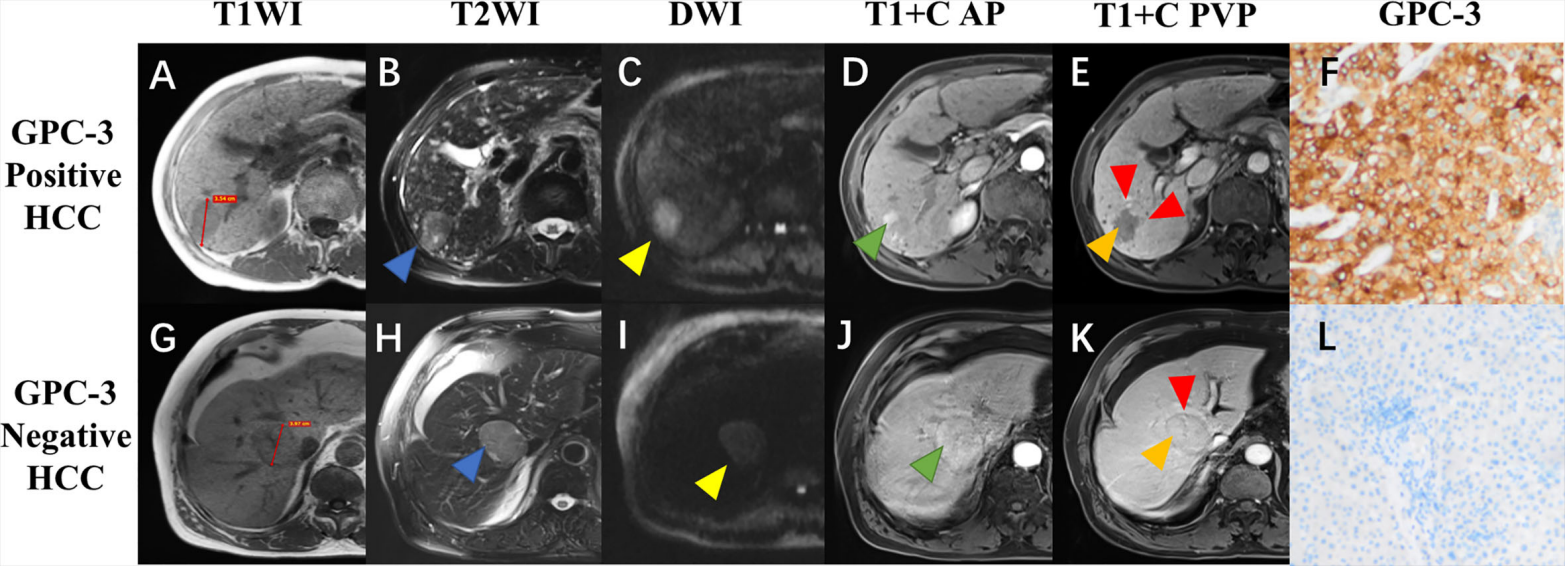
Gadoxetate disodium-enhanced MRI and histopathologic images of hepatocellular carcinoma (HCC) with different glypican-3 (GPC-3) expressions. A 72-year-old male patient with GPC-3 positive expression HCC **(A–F)**, and a 77-year-old male patient with GPC-3 negative expression HCC **(G–L)**. Pre-contrast T1-weighted images showed a hypointense lesion (3.54cm) in the right liver **(A)** and a hypointense lesion (3.97cm) in the mid liver **(G)**; T2-weighted images showed hyperintense lesions with [**(B)**, blue arrow] and without [**(H)**, blue arrow] “iron sparing in solid mass”; diffusion-weighted images demonstrated presence [**(C)**, yellow arrow] and absence [**(I)**, yellow arrow] of “marked diffusion restriction”; arterial phase images showed “nonperipheral-nonglobal arterial phase hyperenhancement” [**(D)**, green arrow) and “global arterial phase hyperenhancement” [**(J)**, green arrow]; portal venous phase images showed “nonperipheral washout” and infiltrative appearance [**(E)**, orange and red arrow] and “no-washout” and smooth margin [**(K)**, orange and red arrow]; immunohistochemical staining revealed the GPC-3 positive **(F)** and negative **(L)** expressions.

On the training dataset, 14 clinical variables and 37 imaging features were significantly associated with GPC-3 expression at univariate analysis; while serum AFP (odds ratio [OR]=2.3, 95% confidence interval [CI]: 1.1-4.7, corresponding to four points in the GPC-3 score for >10ng/mL), tumor size (OR=0.5, 95%CI: 0.2-1.0, corresponding to -3 points in the GPC-3 score for >3.0 cm), nonperipheral “washout” (OR=3.0, 95%CI: 1.3-7.2, corresponding to five points in the GPC-3 score for its presence), infiltrative appearance (OR=9.3, 95% CI: 1.8-46.4, corresponding to 10 points in the GPC-3 score for its presence), marked diffusion restriction (OR=3.3, 95%CI: 1.5-7.4, corresponding to five points in the GPC-3 score for its presence) and iron sparing in solid mass (OR=0.2, 95% CI: 0.1-0.6, corresponding to -7 points in the GPC-3 score for its presence) were significantly associated with GPC-3 expression at multivariate logistic regression analysis (**Figure 2**; **Table 3**).

**Table 3:** The relationships between GPC-3 expression with clinical and MRI features in HCC patients.

	Univariate			Multivariate		
Variables	Coefficient	*P* value	OR (95%CI)	Coefficient	*P* value	OR (95%CI)
Age	-0.02	0.24	1 (0.9-1)	–	–	–
Sex	-0.24	0.58	0.8 (0.3-1.8)	–	–	–
HBV infection	0	1	1 (0.3-3.9)	–	–	–
Cirrhosis	-0.36	0.35	0.7 (0.3-1.5)	–	–	–
AFP (>10 ng/ml vs. ≤10 ng/ml)	0.69	0.06^#^	2 (1-4.2)	0.82	0.03^#^	2.3 (1.1-4.7)
PIVKA.II (>32.5 AU/ml vs. ≤32.5 AU/ml)	0.13	0.75	1.1 (0.5-2.6)	–	–	–
CEA (>3.4 ng/ml vs. ≤3.4 ng/ml)	-0.15	0.73	0.9 (0.4-2.1)	–	–	–
CA199 (>22 U/ml vs. ≤22 U/ml)	-0.31	0.42	0.7 (0.3-1.6)	–	–	–
TBIL (>28 μmol/L vs. ≤28 μmol/L)	0	1	1 (0.3-3.9)	–	–	–
ALT (>50 U/L vs. ≤50 U/L)	-0.77	0.08	0.5 (0.2-1.1)	–	–	–
AST (>40 U/L vs. ≤40 U/L)	-0.94	0.02	0.4 (0.2-0.9)	–	–	–
ALB (>40 U/L vs. ≤40 U/L)	0.64	0.14	1.9 (0.8-4.4)	–	–	–
PLT (>100 ×109/L vs. ≤100 ×109/L)	0.61	0.11	1.8 (0.9-3.9)	–	–	–
Size (>3.0 cm vs. ≤3.0 cm)	-0.78	0.04^#^	0.5 (0.2-1)	-0.77	0.05^#^	0.5 (0.2-1)
APHE	0.25	0.54	1.3 (0.6-2.9)	–	–	–
Internal artery	-0.53	0.18	0.6 (0.3-1.3)	–	–	–
Corona enhancement	0.29	0.48	1.3 (0.6-3)	–	–	–
Nonperipheral “washout”	0.81	0.07^#^	2.3 (0.9-5.3)	1.11	0.01^#^	3 (1.3-7.2)
Complete capsule	0.1	0.78	1.1 (0.5-2.3)	–	–	–
Blood products in mass	-0.04	0.92	1 (0.4-2.1)	–	–	–
Nodule in nodule	-0.28	0.48	0.8 (0.3-1.6)	–	–	–
Mosaic architecture	-0.27	0.49	0.8 (0.4-1.6)	–	–	–
Infiltrative appearance	1.93	0.06^#^	6.9 (0.9-53.6)	2.23	0.01^#^	9.3 (1.8-46.4)
Necrosis or severe ischemia	-0.39	0.34	0.7 (0.3-1.5)	–	–	–
Tumor margin	0.03	0.93	1 (0.5-2.1)	–	–	–
Marked diffusion restriction	0.98	0.02^#^	2.7 (1.2-6.1)	1.2	<0.01^#^	3.3 (1.5-7.4)
Marked T2 hyperintense	15.49	0.99	5354412.9 (0-Inf)	–	–	–
Fat in mass more than liver	0.56	0.14	1.7 (0.8-3.7)	–	–	–
Fat sparing in solid mass	0	1	1 (0.3-3.9)	–	–	–
Iron in mass more than liver	0	1	1 (0.1-9.9)	–	–	–
Iron sparing in solid mass	-0.78	0.09	0.5 (0.2-1.1)	-1.47	<0.01^#^	0.2 (0.1-0.6)
HBP hypointense	1.84	0.14	6.3 (0.6-71.1)	–	–	–
HBP Peritumoral hypointense	0.5	0.21	1.6 (0.7-3.6)	–	–	–
Targetoid	13.48	0.99	712146.7 (0-Inf)	–	–	–

^#^With statistic difference.

CEA, carcinoma embryonic antigen; TBIL, total bilirubin; ALT, alanine transaminase; AST, aspartate aminotransferase; ALB, serum albumin; PLT, platelet count; APHE, arterial phase hyperenhancement; HBP, hepatobiliary phase; OR, odds ratio.

Inter-rater agreement was moderate for nonperipheral “washout” (κ=0.400, 95%CI: 0.307-0.494), infiltrative appearance (κ=0.430, 95%CI: 0.266-0.576), marked diffusion restriction (κ=0.387, 95%CI: 0.261-0.497) and iron sparing in solid mass (κ= 0.432, 95%CI: 0.293-0.561). Inter-rater agreements on the remaining imaging features are summarized in **Table 2**.

### Validation of the GPC-3 prediction model

The AUC for the GPC-3 prediction model was 0.775 (95% CI, 0.694- 0.855), 0.726 (95% CI, 0.558- 0.894) and 0.681 (95% CI, 0.547-0.81) for the training, internal validation, and external validation datasets, respectively (**Table 4**).

**Table 4:** The performance of predictive model for GPC-3 positive expression in HCC patients.

	Internal training set	Internal validation set	External validation set
AUC and 95% CI	0.775 (0.694 - 0.855)	0.726 (0.558 - 0.894)	0.681 (0.547 - 0.814)
Sensitivity and 95% CI	0.829 (74.8% - 89.2%)	0.793 (60.3% - 92%)	0.806 (68.6% - 89.6%)
Specificity and 95% CI	0.564 (39.6% - 72.2%)	0.500 (18.7% - 81.3%)	0.381 (18.1% - 61.6%)
PPV and 95% CI	0.851 (77.2% - 91.1%)	0.821 (63.1% - 93.9%)	0.794 (67.3% - 88.5%)
NPV and 95% CI	0.524 (36.4% - 68.0%)	0.455 (16.7% - 76.6%)	0.400 (19.1% - 63.9%)
ACC and 95% CI	0.763 (68.8% - 82.7%)	0.718 (55.1% - 85.0%)	0.699 (58.8% - 79.5%)

AUC, area under the curve; 95% CI, 95% confidence interval; PPV, positive predictive value; NPV, negative predictive value; ACC, accuracy.

According to Youden’s index, the optimal threshold of scoring system for predicting GPC-3 expression was 5.5 points, and patients with the GPC-3 scores ≥5.5 points were categorized as at high-risk for positive GPC-3 expression in HCC. Based on this threshold, the sensitivity, specificity, PPV, NPV, and accuracy of the GPC-3 score on the internal validation dataset was 50.7% (95% CI, 42.3%-59%), 87.8% (95% CI, 75.2%-95.4%), 92.5% (95% CI, 84.4%-97.2%), 37.4% (95% CI, 28.5%-46.9%), and 60% (95% CI, 52.8%-66.9%), respectively. On the external validation dataset, these measures were 43.5% (95% CI, 31%-56.7%), 81.0% (95% CI, 58.1%-94.6%), 87.1% (95% CI, 70.2%-96.4%), 32.7% (95% CI, 20.3%-47.1%), and 53% (95% CI, 41.7%-64.1%), respectively.

**Figure 3:**
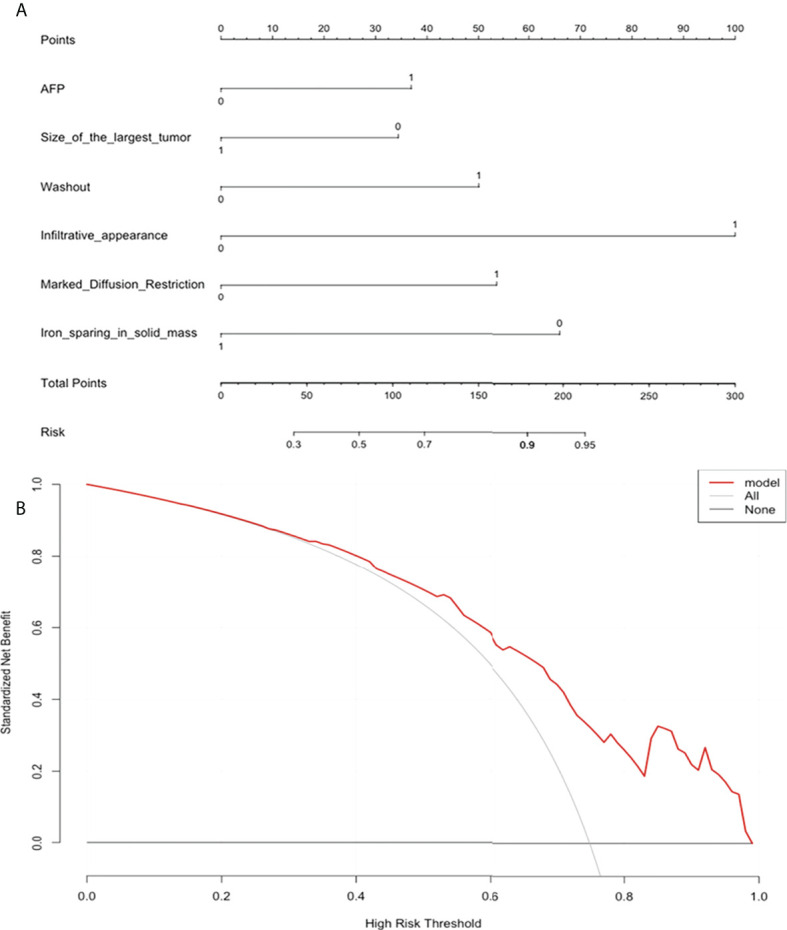
The nomogram and decision curve to predict glypican-3 positive expression in hepatocellular carcinoma. **(A)** The nomogram was developed based on serum alpha-fetoprotein and five MRI features (tumor size, nonperipheral “washout”, infiltrative appearance, marked diffusion restriction, and iron sparing in solid mass). **(B)** Decision curve analysis of the prediction model for external validation set. Y-axis represents the net benefit, which is calculated by gaining true positives and deleting false positives. The X-axis is the probability threshold. The curve of the predictive model over the AFP and MRI features that showed the good benefit.

**Figure 4:**
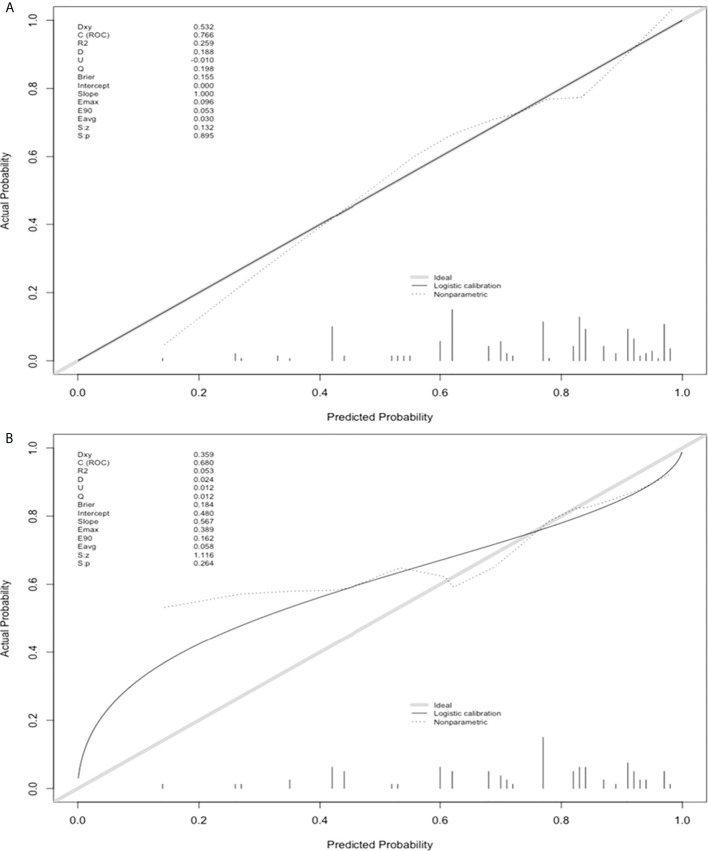
Model calibration curves on the training **(A)** and validation sets **(B)** to predict glypican-3 positive expression in hepatocellular carcinoma.

The nomogram and decision curves revealed substantial clinical benefit of the prediction model in predicting GPC-3 expression (**Figure 3**). The calibration curves showed good agreement between predicted and observed probabilities of positive GPC-3 expression for both the training (*P*=0.895) and validation (*P*=0.264) datasets (**Figure 4**).

### Survival analysis

**Figure 5:**
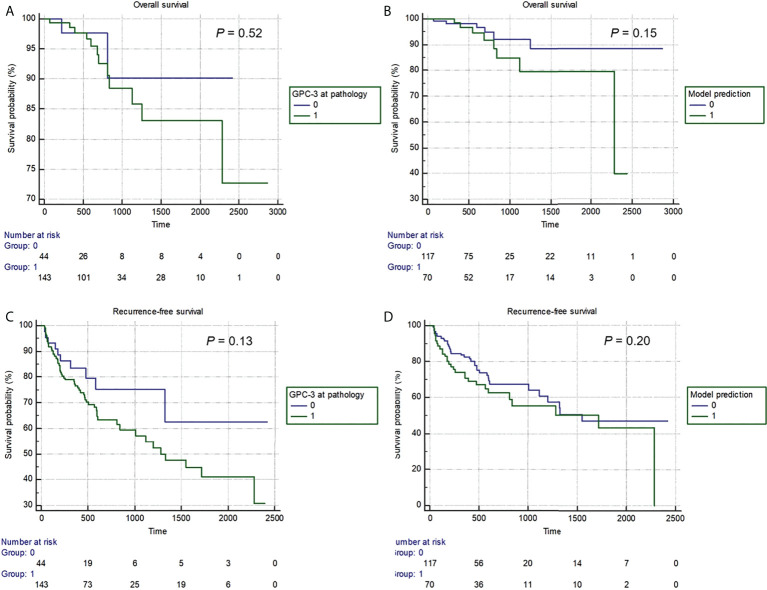
The Kaplan-Meier curves of pathologically-confirmed **(A, C)** and model-predicted glypican-3 expressions **(B, D)** in stratifying postoperative overall survival **(A, B)** and recurrence-free survival **(B, D)**.

A total of 187 (62%) patients had complete RFS and OS information, thus were included in the survival analyses. Median follow-up was 668 days (95% CI: 589-734 days). Among them, 14 (7%) patients died, and 66 (35%) patients experienced tumor recurrence. Median OS was 1613 days (95% CI: 972-1973 days), while median RFS was 1548 days (95% CI: 1113-2281 days). No difference in RFS and OS was detected between patients with pathologically or model-predicted confirmed GPC-3 expressions. (*P* values= 0.13, 0.20, 0.52 and 0.150, respectively), despite a trend-wise longer survival in patients with GPC-3 negative HCC (**Figure 5**).

## Discussion

In patients who underwent curative-intent liver resection for solitary HCC, we developed and validated a noninvasive risk score to predict GPC-3 expression based on serum AFP and five EOB-MRI features (tumor size, nonperipheral “washout”, infiltrative appearance, marked diffusion restriction, and iron sparing in solid mass). On the independent validation testing set, the prediction model demonstrated moderate diagnostic performance and gool calibration for GPC-3 expression.

Previous studies have revealed that GPC-3 is actively involved in regulating HCC tumor growth (22, 23). Shirakawa et al. (24) reported that positive GPC-3 expression is correlated with poor clinical prognosis for HCC patients, and Li et al. (25) illustrated that GPC-3 is a coreceptor with the activation of the Wnt signaling pathway. The authors considered it to be a potential target site for HCC therapy that restrains Wnt signaling. Currently, novel treatments for HCC have been explored and assessed in *in vitro* and *in vivo* experiments and clinical trials targeting GPC-3, such as CAR T cell therapy (26), immunotoxin therapy (27), and GPC-3-derived peptide vaccines (28). Therefore, it is of great significance to evaluate the expression of GPC-3 in HCC preoperatively, noninvasively and precisely. Chen et al. (19) reported that the R2* value yielded from iterative decomposition of water and fat with echo asymmetry and least squares estimation MRI could reliably predict GPC-3 expression in HCC prior to surgery. In addition, Gu et al. (18) demonstrated that the proposed MR-based radiomics signature is strongly related to GPC-3 positivity and incorporating AFP levels and radiomics signatures may noninvasively and individually predict GPC-3-positive HCC. In our study, 278 solitary patients were included who underwent hepatectomy, and postoperative pathology assessed GPC-3 expression. A total of 208 (74.8%) of the HCC lesions were GPC-3 positive, and 70 (25.2%) were negative. The results revealed that AFP>10 ng/ml, tumor size>3.0 cm, nonperipheral “washout”, infiltrative appearance, marked diffusion restriction, and iron sparing in solid mass were significantly associated with GPC-3 positive expression. Chu et al. (29) illustrated a GPC3-based immunomagnetic fluorescent system (C6/MMSN-GPC3) that showed the high-specific isolation and instant observation of HCC circulating tumor cells. But in our study, we directly assessed the GPC-3 expression in HCC tumor tissues by MRI features which no need peripheral blood and without radiation.

The liver imaging reporting and data system (LI-RADS) is supported by the American College of Radiology (https://www.acr.org) and provides standardization of liver imaging and reporting. It offers four individual imaging algorithms designed for different clinical contexts of HCC risk patients, which include ultrasound LI-RADS for surveillance, contrast-enhanced US, CT or MRI LI-RADS for diagnosis and staging, and treatment response LI-RADS to assess response to local-regional therapies. All LI-RADS algorithms are built on the foundation of standardized lexicon, technique, management, and reporting guidelines (30). LI-RADS assigns features that reflect the probability of HCC, nonHCC malignancy, or benignity (31), assesses the association between LI-RADS categories with microvascular invasion (MVI) and histologic grade of HCC (32), predicts the recurrence of HCC after primary liver transplantation within the Milan criteria (33), and evaluates the molecular alterations during hepatocarcinogenesis (34). Zhao et al. (17) enrolled 43 and 100 patients with pathologically confirmed GPC-3 negative and positive patients with contrast-enhanced MRI and diffusion-weighted imaging (DWI) to explore the potential MRI findings in predicting GPC-3 positive HCCs. The results showed that the serum AFP levels and lower 75th percentile apparent diffusion coefficient (ADC) values were helpful in differentiating GPC-3 positive HCCs. However, the 75th percentile ADC value was not included in clinical LI-RADS analysis; in addition to requiring professional software measurement, it has disadvantages such as poor interpretability and inconveniences of clinical application. Therefore, our study comprehensively analyzed the differential ability of LI-RADS MRI features and categories for GPC-3-positive HCC that has not been previously reported in the literature.

After univariate and multivariate regression analyses, AFP, tumor size, nonperipheral “washout”, infiltrative appearance, marked diffusion restriction, and iron sparing in solid mass were selected to construct the prediction model of this study. Based on the 5.5 points threshold, our model demonstrated a specificity of 87.8% in predicting positive GPC-3 expression in HCC. Therefore, considering remarkable incidence of adverse effects and high cost of HCC immunotherapy, our model may help avoid inappropriate treatment initiation, minimize unwanted adverse effect, reduce unnecessary cost, and improve individualized treatment decision-making. AFP is a progenitor cell marker that has been considered useful in the early screening and assisting diagnosis of HCC and as a biomarker helping select candidates in testing drugs in Phase III trials or evaluating curative effects (35, 36). Ye et al. illustrated that patients with high serum AFP levels and GPC-3 positive expression were associated with a poor prognosis (37). Our results showed that there was a positive association between serum AFP levels >10 ng/ml with GPC-3 positive expression.

Nonperipheral “washout” appearance is an important major imaging feature of LI-RADS, it is a visually assessed temporal reduction in enhancement of an observation relative to composite liver tissue from an earlier to a later phase resulting in hypoenhancement on the portal venous (PVP) or delayed phases (38), Kim et al. (39) revealed that nonperipheral “washout” appearance on the PVP or transitional phase is the most reliable MR imaging feature for differentiating hepatocellular carcinoma with paradoxical uptake on the hepatobiliary phase from focal nodular hyperplasia-like nodules. Additionally, our study showed that the nonperipheral “washout” feature is an independent risk factor for evaluating HCC with positive GPC-3. The infiltrative appearance may represent true infiltration of tumor cells into the liver parenchyma, which commonly indicates malignancy with a permeative growth pattern and is associated with macrovascular invasion (40), tumor metastasis and a short survival time (41), Sun et al. (42) reported that the infiltrative tumor margin has the potential to identify PD-L1-positive HCC, similarly our results demonstrated that this MR imaging feature also hints at GPC-3 positive expression. Tumor size is also included as a major feature of LI-RADS since the likelihood of malignancy in a cirrhosis-associated nodule is positively correlated with the size of the observation (43), but the tumor size exceeding 3.0 cm was considered to be more likely associated with negative GPC-3 expression in our study.

Diffusion restriction is an ancillary MR imaging feature favoring malignancy in general. The minimum apparent diffusion coefficient is a significantly independent risk factor for early HCC recurrence after surgery (44), and Joo et al. (45) showed that the diffusion restriction feature was an independent predictor to help differentiate progressed HCC from low- or high-grade nodules because the expression of GPC-3 in HCC is obviously higher than that in low- or high-grade nodules; thus, the finding may confirm our results that marked diffusion restriction is also an independent predictor for GPC-3 positive expression. Also, iron sparing in solid masses is an ancillary LI-RADS feature that usually favors liver malignancy and is defined as a paucity of iron in solid masses relative to iron-overloaded livers or in inner nodules relative to siderotic outer nodules, a paucity of iron suggests clonal expansion of a cell with iron resistance distinct from the background parenchyma (46), the results of our study showed that this LI-RADS feature strongly indicates the GPC-3 negative expression in HCC, which may indicate that malignant hepatic cells with reduced iron intake is more likely to be accompanied by GPC-3 negative expression.

In this study, during the follow-up, 14 (7%) patients died, and 66 (35%) patients experienced tumor recurrence. Despite no significant difference in RFS or OS detected between patients with pathologically or model-predicted confirmed GPC-3 expression, Kaplan–Meier survival curves still showed a trend wise longer survival in patients with GPC-3-negative HCC. The possible reason is that the follow-up time was not long enough, which led to the small number of patients with OS and RFS events, only 7% and 35%, respectively. Nevertheless, this study also suggests that GPC-3 expression is of great significance for the prognosis of HCC patients.

Several limitations in this study should be noted. First, the retrospective nature could have introduced selection biases to the study cohort, and in this predictive model was developed in patients with solitary HCCs to improve radiology-pathology correlations. However, this design also hampered the extrapolation of our findings to multiple tumors. Therefore, future studies are encouraged to explore the correlation between MRI features and GPC-3 expressions in multiple tumors while guaranteeing rigorous radiology-pathology spatial correlation. Second, the number of enrolled patients with GPC-3-negative HCC was relatively small (70 patients [25.2%]), and the imbalanced sample size may have reduced the effectiveness of model training. However, this was in part because we only analyzed data from patients with solitary HCC. This was determined to improve radiology-pathology correlation and to minimize the interference of confounding factors. Third, although CAR T cell targeting GPC-3 therapy for advanced HCC patients is considered next-generation immunotherapy, our study did not directly evaluate the efficacy of CAR T cell therapy. Nevertheless, this study constructed an easy-to-use predictive model that could be used for noninvasive assessment for GPC-3 expression in HCC, which may provide useful information regarding patient selection for future GPC-3-targeted immunotherapy. Fourth, at present, we only established the model based on the qualitative features of EOB-MRI, although it has the advantages of simple operation and strong interpretation, but it does not use any functional/molecular imaging techniques. Other molecular imaging techniques, for example, positron emission tomography-computed tomography and single-photon emission computed tomography-computed tomography, allow direct depiction for the expression of tumor-specific targets, thus may serve as promising noninvasive tools for GPC-3 expression assessment in HCC (47).

In conclusion, in patients with surgically-confirmed solitary HCC, we developed and validated an easy-to-use noninvasive prediction model which could accurately predict positive GPC-3 HCC based on serum AFP and five EOB-MR imaging features in HCC patients. Our findings may help identify potential responders for GPC-3-targeted immunotherapy and direct personalized treatment decision-making.

## Data availability statement

The original contributions presented in the study are included in the article/**Supplementary Material**. Further inquiries can be directed to the corresponding authors.

## Ethics statement

The studies involving human participants were reviewed and approved by the ethics committee of West China Hospital, Sichuan University. Written informed consent for participation was not required for this study in accordance with the national legislation and the institutional requirements.

## Author contributions

YC, YQ and HJ conceived of and designed this study. ZZ and TD performed the experiments. YNW and HJ performed the data analysis. BS and YW reviewed this manuscript. All authors contributed to the article and approved the submitted version.

## Funding

This work was funded by the National Natural Science Foundation of China (Grant No. 82101997, 81971571) and the Science and Technology Department of Sichuan Province (Grant No. 2022YFS0071, 2021YFS0021, 2021YFS0141).

## Conflict of interest

The authors declare that the research was conducted in the absence of any commercial or financial relationships that could be construed as a potential conflict of interest.

## Publisher’s note

All claims expressed in this article are solely those of the authors and do not necessarily represent those of their affiliated organizations, or those of the publisher, the editors and the reviewers. Any product that may be evaluated in this article, or claim that may be made by its manufacturer, is not guaranteed or endorsed by the publisher.
