# Supplementary material for: Preoperative prediction of glypican-3 positive expression in solitary hepatocellular carcinoma on gadoxetate-disodium enhanced magnetic resonance imaging
Source: Front Immunol. 2022 Aug 25;13:973153. doi: 10.3389/fimmu.2022.973153 (PMC9453305; doi:10.3389/fimmu.2022.973153)
Supplement: Supplementary file 1 [file DataSheet_1.pdf]

Preoperative prediction of glypican-3 positive expression in solitary hepatocellular carcinoma on gadoxetate-disodium enhanced Magnetic Resonance Imaging

Supplementary Material

Supplementary A1

MRI technique

MRI examinations were performed with four 3.0-T systems (MAGNETOM Skyra, Siemens Healthineers; Discovery MR 750, GE Healthcare; SIGNA™ Architect, GE Healthcare; and SIGNA™ Premier, GE Healthcare) and a 1.5-T system (uMR588, United Imaging Healthcare). The MRI sequences included T2-weighted two-dimensional fast spin echo imaging, diffusion-weighted imaging (b values: 0, 50, 500, 800, 1000, and 1200 s/mm^2 [Siemens MAGNETOM Skyra]; 0, 200, 800, and 1000 s/mm^2 [GE Discovery MR 750]; 0, 50, and 800 s/mm^2 [GE SIGNA™ Architect 3.0 Tesla]; 50 and 1000 s/mm^2 [GE SIGNA™ Premier 3.0 Tesla]; and 0, 50, and 1000 s/mm^2 [uMR588 1.5 Tesla]) with apparent diffusion coefficient (ADC) maps, T1-weighted dual gradient-echo in- and opposed-phase imaging, and dynamic T1- weighted three-dimensional gradient-echo imaging before and after injection of gadoxetic acid disodium (Primovist®, Bayer Pharma AG) in the late arterial phase, portal venous phase (60 s), transitional phase (3 minutes), and hepatobiliary phase (20 minutes). The arterial phase images were determined either by the acquisition triggered 7 s after the arrival of the contrast bolus in the celiac trunk or by a multiple arterial phase (MAP) imaging technique. Specifically, the MAP images were acquired with an 18 s breath hold 20 s following the contrast agent injection and further reconstructed with a temporal resolution of 3 s. For dynamic imaging, the contrast agent was injected at a rate of 1-2 ml/s for a total dose of 0.025 mmol/kg body weight, followed by 20-30 ml of 0.9% saline flush. Details of the MRI sequences and parameters are shown in **Table S1.**

Table S1 MRI sequences and parameters in our institution

Sequence	T1-weighted IP and OP imaging	Dynamic T1-weighted 3D GRE	T2-weighted 2D FSE	Diffusion-weighted imaging†
**Siemens MAGNETOM Skyra (18-channel body array coil)**				
Repetition time (ms)	81	3.95	2160	5600
Echo time (ms)	2.72/1.4	1.92	100	68
Flip angle (°)	70	9	160	90
Section thickness (mm)	6	2.5	6	6
Spacing (mm)	1.8	-	1.8	1.8
Matrix size	352×286	352×256	320×288	100×76
Field of view (mm^2)	400×325	400×296	433×433	380×289
Acquisition time (s)	24	14	36	233
Fat suppression	No	Yes	Yes	Yes
**GE Discovery MR 750 (16-channel phased-array torsor coil)**				
Repetition time (ms)	150	4.1	6315	9230
Echo time (ms)	2.5/1.3	1.9	78	Minimum
Flip angle (°)	70	15	111	90
Section thickness (mm)	6	2	6	6
Spacing (mm)	2	-	2	2
Matrix size	288×192	512×512	288×244	128 × 128
Field of view (mm^2)	420×420	380× 300	360×280	360× 380
Acquisition time (s)	31	15	RG	RG
Fat suppression	No	Yes	Yes	Yes
**GE SIGNA™ Architect 3.0 Tesla (30-channel body anterior coil)**				
Repetition time (ms)	233.8	3.9	2400	5000
Echo time (ms)	2.3/1.1	1.7	85	Minimum
Flip angle (°)	55	15	111	90
Section thickness (mm)	7	3	7	7
Spacing (mm)	2	-	2	2
Matrix size	160×288	320×240	320×192	160×128
Field of view (mm^2)	380×323	380×380	380×304	380×342
Acquisition time (s)	18	15	34	RG
Fat suppression	No	Yes	Yes	Yes
**GE SIGNA™ Premier 3.0 Tesla (30-channel body anterior coil)**				
Repetition time (ms)	146.8	3.2	2200	5000
Echo time (ms)	2.3/1.1	1.4	85	Minimum
Flip angle (°)	55	15	111	90
Section thickness (mm)	7	2.4	7	7
Spacing (mm)	2	-	2	2
Matrix size	320×192	320×240	320×224	120 × 240
Field of view (mm^2)	342×380	380× 380	304×380	380× 380
Acquisition time (s)	16	15	47	RG

Fat suppression	No	Yes	Yes	Yes
**uMR588 1.5 Tesla (6-channel body anterior coil)**				
Repetition time (ms)	117.6	4.2	2600	3350
Echo time (ms)	2.27	1.88	99.2	77
Flip angle (°)	60	10	90	90
Section thickness (mm)	6.5	2.5	6.5	6.5
Spacing (mm)	1.3	-	1.5	10
Matrix size	256×174	256×154	256×168	128×92
Field of view (mm^2)	320×400	255×400	427×320	320×400
Acquisition time (s)	29	13	39	RG
Fat suppression	No	Yes	Yes	Yes

MRI, magnetic resonance imaging; IP, in-phase; OP, opposed-phase; 3D, three-dimensional; GRE, gradient recall echo; 2D, two-dimensional; FSE, fast spin-echo; RG, respiratory gating.

[†]Images were acquired under free breath.

Table S2 Definitions of the evaluated EOB-MR imaging features (which based on CT/MRI Liver Imaging Reporting and Data System version 2018)

MRI feature	Definition
**Tumor diameter or Size**	Largest outer-edge-to-outer-edge dimension of a liver observation
**Nonrim arterial phase hyperenhancement**	Nonrim-like enhancement of the liver observation in arterial phase unequivocally greater in whole or in part than liver
**Nonperipheral "washout"**	Nonperipheral visually assessed temporal reduction in enhancement of the liver observation in whole or in part relative to composite liver tissue from earlier to later phase resulting in hypoenhancement in the extracellular phase
**Enhancing "capsule"**	Smooth, uniform, sharp border around most or all of a liver observation, unequivocally thicker or more conspicuous than fibrotic tissue around background nodules, and visible as enhancing rim in portal venous phase or transitional phase
**Corona enhancement**	Periobservational enhancement in late arterial phase or early portal venous phase attributable to venous drainage from tumor
**Fat sparing in solid mass**	Relative paucity of fat in solid mass relative to steatotic liver OR in inner nodule relative to steatotic outer nodule
**Restricted diffusion**	Signal intensity of the liver observation on diffusion-weighted imaging, not attributable solely to T2 shine-through, unequivocally higher than liver and/or apparent diffusion coefficient unequivocally lower than liver
**Mild-moderate T2 hyperintensity**	Signal intensity of the liver observation on T2-weighted imaging mildly or moderately higher than liver and similar to or less than non-iron-overloaded spleen
**Iron sparing in solid mass**	Paucity of iron in solid mass relative to iron-overloaded liver OR in inner nodule relative to siderotic outer nodule
**Transitional phase hypointensity**	Signal intensity of the liver observation in the transitional phase unequivocally less, in whole or in part, than liver
**Hepatobiliary phase hypointensity**	Signal intensity of the liver observation in the hepatobiliary phase unequivocally less, in whole or in part, than liver
**Non-enhancing "capsule"**	Capsule appearance not visible as an enhancing rim

**Nodule-in-nodule architecture**	Presence of smaller inner nodule within and having different imaging features than larger outer nodule
**Mosaic architecture**	Presence of randomly distributed internal nodules or compartments, usually with different imaging features
**Fat in mass, more than adjacent liver**	Excess fat within a mass, in whole or in part, relative to adjacent liver
**Blood products in mass**	Intralesional or perilesional hemorrhage in the absence of biopsy, trauma or intervention
**Parallels blood pool enhancement**	Temporal pattern in which enhancement eventually reaches and then matches that of blood pool
**Undistorted vessels**	Vessels traversing an observation without displacement, deformation, or other alteration
**Iron in mass, more than liver**	Excess iron in a mass relative to background liver
**Marked T2 hyperintensity**	Signal intensity of the liver observation on T2 weighed-imaging markedly higher than liver and similar to bile ducts and other fluid-filled structures
**Hepatobiliary phase isointensity**	Signal intensity of the liver observation in the hepatobiliary phase nearly identical to liver
**Tumor in vein**	Presence of unequivocal enhancing soft tissue in vein
**Rim arterial phase hyperenhancement**	Presence of arterial phase enhancement most pronounced in observation periphery
**Peripheral "washout"**	Presence of apparent washout most pronounced in observation periphery
**Delayed central enhancement**	Central area of progressive postarterial phase enhancement
**Targetoid restriction**	Concentric pattern on diffusion-weighted imaging characterized by restricted diffusion in observation periphery with less restricted diffusion in observation center
**Targetoid TP or HBP appearance**	Concentric pattern in TP or HBP characterized by moderate-to-marked hypointensity in observation periphery with milder hypointensity in center
**Infiltrative appearance**	Liver observation with non-circumscribed margin (indistinct transition)
**Marked diffusion restriction**	Increased signal intensity of the liver observation at diffusion-weighted imaging in relative to the spleen, not solely attributable to T2-weighted imaging shine-through effect
**Necrosis or severe ischemia**	Presence of unequivocal intralesional necrosis or

	severe ischemia
**Liver surface retraction**	Presence of focal flattening or concavity of the normally convex hepatic contour
**Adjacent biliary dilatation**	Presence of dilated bile ducts adjacent to the tumor border in any imaging plane
**Radiologic cirrhosis**	An irregular, nodular or shrunken liver, as well as ascites or evidence of portosystemic collaterals in decompensated stage
**Bilobar involvement**	Bilobar involvement of definite HCC on gadoxetic acid-enhanced MR imaging
**Internal artery**	Presence of discrete arterial enhancement within the tumor
**Non-smooth tumor margin**	Presence of non-nodular tumors with irregular contour that had budding portion at the periphery [8]
**Peritumoral hypointensity on HBP**	Presence of wedge-shaped or flame-like hypointense area adjacent to the tumor border on hepatobiliary phase images
**Tumor capsule**	-Complete: Presence of non-disrupted "capsule" in all imaging planes;<br>-Incomplete: Presence of disrupted "capsule" in all imaging planes

EOB-MRI, gadoxetic acid–enhanced magnetic resonance imaging; HCC, hepatocellular carcinoma; PVP, portal venous phase; HBP, hepatobiliary phase; AP, arterial phase; TP, transitional phase; IP, in-phase; OP, opposed-phase; DWI, diffusion-weighted imaging; T2WI, T2-weighted imaging; T1WI, T1-weighted imaging; LI-RADS/LR, Liver Imaging Reporting and Data System.

References

1. CT/MRI Liver Imaging Reporting and Data System version 2018 (2018) Available via https://www.acr.org/Clinical-Resources/Reporting-and-Data-Systems/LI-RADS/CT-MRI-LI-RADS-v2018. Accessed 25 Oct 2021
2. Blachar A, Federle MP, Sosna J (2009) Liver lesions with hepatic capsular retraction. Semin Ultrasound CT MR 30:426-435

3. Ji GW, Zhu FP, Xu Q et al (2020) Radiomic features at contrast-enhanced CT predict recurrence in early stage hepatocellular carcinoma: a multi-institutional study. Radiology 294:568-579
4. Renzulli M, Brocchi S, Cucchetti A et al (2016) Can current preoperative imaging be used to detect microvascular invasion of hepatocellular carcinoma? Radiology 279:432-442
5. Lee S, Kim SH, Lee JE, Sinn DH, Park CK (2017) Preoperative gadoxetic acid-enhanced MRI for predicting microvascular invasion in patients with single hepatocellular carcinoma. J Hepatol 67:526-534
6. Lei Z, Li J, Wu D et al (2016) Nomogram for preoperative estimation of microvascular invasion risk in hepatitis B virus-related hepatocellular carcinoma within the Milan criteria. JAMA Surg 151:356-363

Table S3. baseline clinical and EOB-MRI features among the training and validation datasets

Variables	Training dataset (n=195)	Validation dataset (n=83)	*P* value
Age (year)	53.5±11.7	53.8±11.6	0.874
Sex (n, %)			0.123
Male	151 (77.4%)	71 (85.5%)	
Female	44 (22.6%)	12 (14.5%)	
HBV infection			0.717
Presence	181 (92.8%)	76 (91.6%)	
Absence	14 (7.2%)	7 (8.4%)	
Cirrhosis			0.986
Presence	113 (57.9%)	48 (57.8%)	
Absence	82 (42.1%)	35 (42.2%)	
BCLC A stage			0.796
Presence	130 (66.7%)	54 (65.1%)	
Absence	65 (33.3%)	29 (34.9%)	
AFP (ng/mL)			0.515
≤10	81 (41.5%)	31 (37.3%)	
> 10	114 (58.5%)	52 (62.7%)	
PIVKA.II (AU/ml)			0.509
≤32.5	52 (26.7%)	19 (22.9%)	
> 32.5	143 (73.3%)	64 (77.1%)	
CEA (ng/ml)			0.803
≤3.4	153 (78.5%)	64 (77.1%)	
> 3.4	42 (21.5%)	19 (22.9%)	
CA199 (U/ml)			0.358
≤22	131 (67.2%)	51 (61.4%)	
> 22	64 (32.8%)	32 (38.6%)	
TBIL (μmol/L)			0.727
≤28	181 (92.8%)	78 (94.0%)	
> 28	14 (7.2%)	5 (6.0%)	
ALT (U/L)			0.709
≤50	152 (77.9%)	63 (75.9%)	
> 50	43 (22.1%)	20 (24.1%)	
AST (U/L)			0.933
≤40	140 (71.8%)	60 (72.3%)	
> 40	55 (28.2%)	23 (27.7%)	
ALB (U/L)			0.422
≤40	43 (22.1%)	22 (26.5%)	
> 40	152 (77.9%)	61 (73.5%)	
PLT (10^9/L)			0.904
≤100	62 (31.8%)	27 (32.5%)	
> 100	133 (68.2%)	56 (67.5%)	

GPC-3			0.976
Positive	146 (74.9%)	62 (74.7%)	
Negative	49 (25.1%)	21 (25.3%)	
LI_RADS			0.146
5	152（77.9%)	71（85.5%)	
Other category	43（22.1%)	12（14.5%)	
Size（cm）			0.149
≤3.0	92（47.2%)	47（56.6%)	
> 3.0	103（52.8%)	83（43.4%)	
Nonperipheral nonglobal APHE			0.627
Presence	135（69.2%)	55（66.3%)	
Absence	60（30.8%)	28（33.7%)	
Internal artery			0.055
Presence	54（27.7%)	14（16.9%)	
Absence	141（72.3%)	69（83.1%)	
Corona enhancement			0.476
Presence	60（30.8%)	22（26.5%)	
Absence	135（69.2%)	61（73.5%)	
Nonperipheral nonglobal washout			0.991
Presence	127 (65.1%)	54 (65.1%)	
Absence	68（34.9%)	29（34.9%)	
Complete capsule			0.417
Presence	86（44.1%)	41（49.4%)	
Absence	109（55.9%)	42（50.6%)	
Blood products in mass			1.00
Presence	58（27.9%)	19（27.1%)	
Absence	150（72.1%)	51（72.9%)	
Nodule in nodule			0.144
Presence	59（30.3%)	18（21.7%)	
Absence	136（69.7%)	65（78.3%)	
Mosaic architecture			0.198
Presence	62（31.8%)	20（24.1%)	
Absence	133（68.2%)	63（75.9%)	
Infiltrative appearance			0.175
Presence	25（12.8%)	6（7.2%)	
Absence	170（87.2%)	77（92.8%)	
Necrosis or severe ischemia			0.927
Presence	48（24.6%)	20（24.1%)	
Absence	147（75.4%)	63（75.9%)	
Tumor margin			0.333
Smooth	91（46.7%)	44（53.0%)	
Non-smooth	104（53.3%)	39（47.0%)	
Marked diffusion restriction			0.056
Presence	80（41.0%)	24（28.9%)	

Absence	115（59.0%）	59（77.1%）	
Marked T2 hyperintense			0.326
Presence	5（2.6%）	0（0.0%）	
Absence	190（97.4%）	83（100.0%）	
Fat in mass more than liver			0.341
Presence	92（47.2%）	34（41.0%）	
Absence	103（52.8%）	49（50.9%）	
Fat sparing in solid mass			0.132
Presence	17（8.7%）	3（3.6%）	
Absence	178（91.3%）	80（96.4%）	
Iron in mass more than liver			0.678
Presence	6（3.1%）	1（1.2%）	
Absence	189（96.9%）	82（92.8%）	
Iron sparing in solid mass			0.873
Presence	36（18.5%）	16（19.3%）	
Absence	159（81.5%）	67（80.7%）	
HBP hypointense			0.313
Presence	189（96.9%）	78（94.0%）	
Absence	6（3.1%）	5（6.0%）	
HBP Peritumoral hypointense			0.013
Presence	72（36.9%）	18（21.7%）	
Absence	123（63.1%）	65（78.3%）	
Targetoid TP or HBP appearance			1.00
Presence	2（1.0%）	0（0.0%）	
Absence	193（99.0%）	83（100%）	

Data are expressed as n (%).

EOB-MRI, gadoxetic acid–enhanced magnetic resonance imaging; LI-RADS/LR, Liver Imaging Reporting and Data System; APHE, arterial phase hyperenhancement; TP, transitional phase; HBP, hepatobiliary phase.

Supplementary A2

Assessment of our proposed model in patients with small or large tumors

In response to the comment, we evaluated the diagnostic performances of our proposed GPC-3 prediction model separately in solitary large (> 3cm) and small (≤3cm) HCCs, respectively. The AUC of the GPC-3 positive expression was 0.771 (Supplementary figure 1) and 0.739 (Supplementary figure 2) for large and small HCCs, respectively. These results revealed comparably satisfactory diagnostic performances of our proposed model for HCCs with different size ranges.

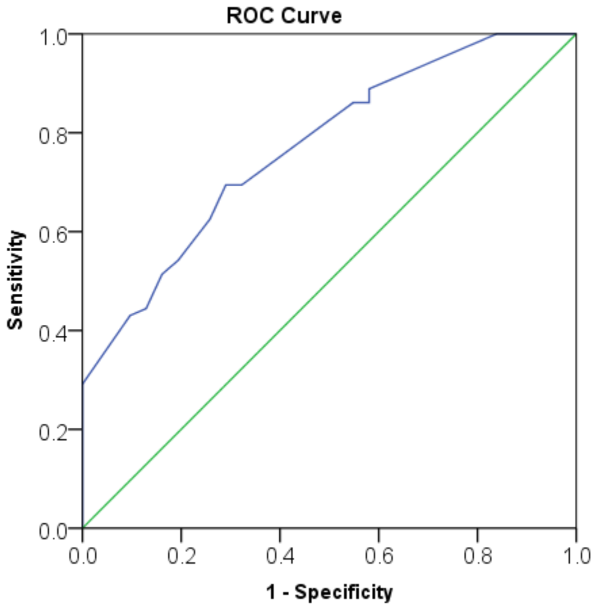

Supplementary figure 1. Receiver operating curve of model prediction probability for GPC-3 expression in large solitary hepatocellular carcinoma (> 3cm), which demonstrates an AUC of 0.771 (95%CI: 0.678 - 0.848, P<0.0001). Based on the optimal threshold value of the model prediction probability, the sensitivity, specificity, positive predictive value, and negative predictive value was 69.44% (95%CI: 57.5 - 79.8%), 71.0% (95%CI: 52.0 - 85.8%), 84.7% (95%CI: 72.9 - 92.8%), and, 50.0% (95%CI: 34.6 - 65.4%), respectively.

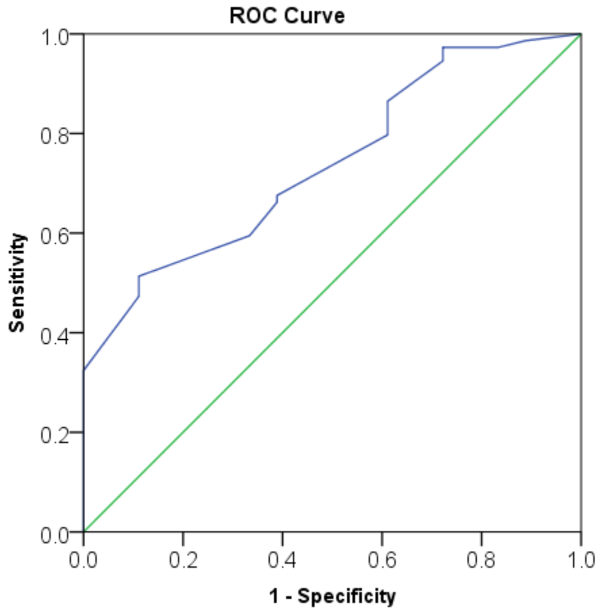

Supplementary figure 2. Receiver operating curve of model prediction probability for GPC-3 expression in large solitary hepatocellular carcinoma (≤ 3cm), which demonstrates an AUC of 0.739 (95%CI: 0.638 - 0.825, P<0.0001). Based on the optimal threshold value of the model prediction probability, the sensitivity, specificity, positive predictive value, and negative predictive value was 51.35% (95%CI: 39.4 - 63.1%), 88.89% (95%CI: 65.3 - 98.6%), 95.0% (95%CI: 83.1 - 99.4%), and, 30.8% (95%CI: 18.6 - 45.3%), respectively.
